# Supplementary figures and images for: Developmental constraints in the repeated evolution of male tail characters in rhabditid and diplogastrid nematodes
Source: PLoS One. 2026 Apr 28;21(4):e0348186. doi: 10.1371/journal.pone.0348186 (PMC13123947; doi:10.1371/journal.pone.0348186)

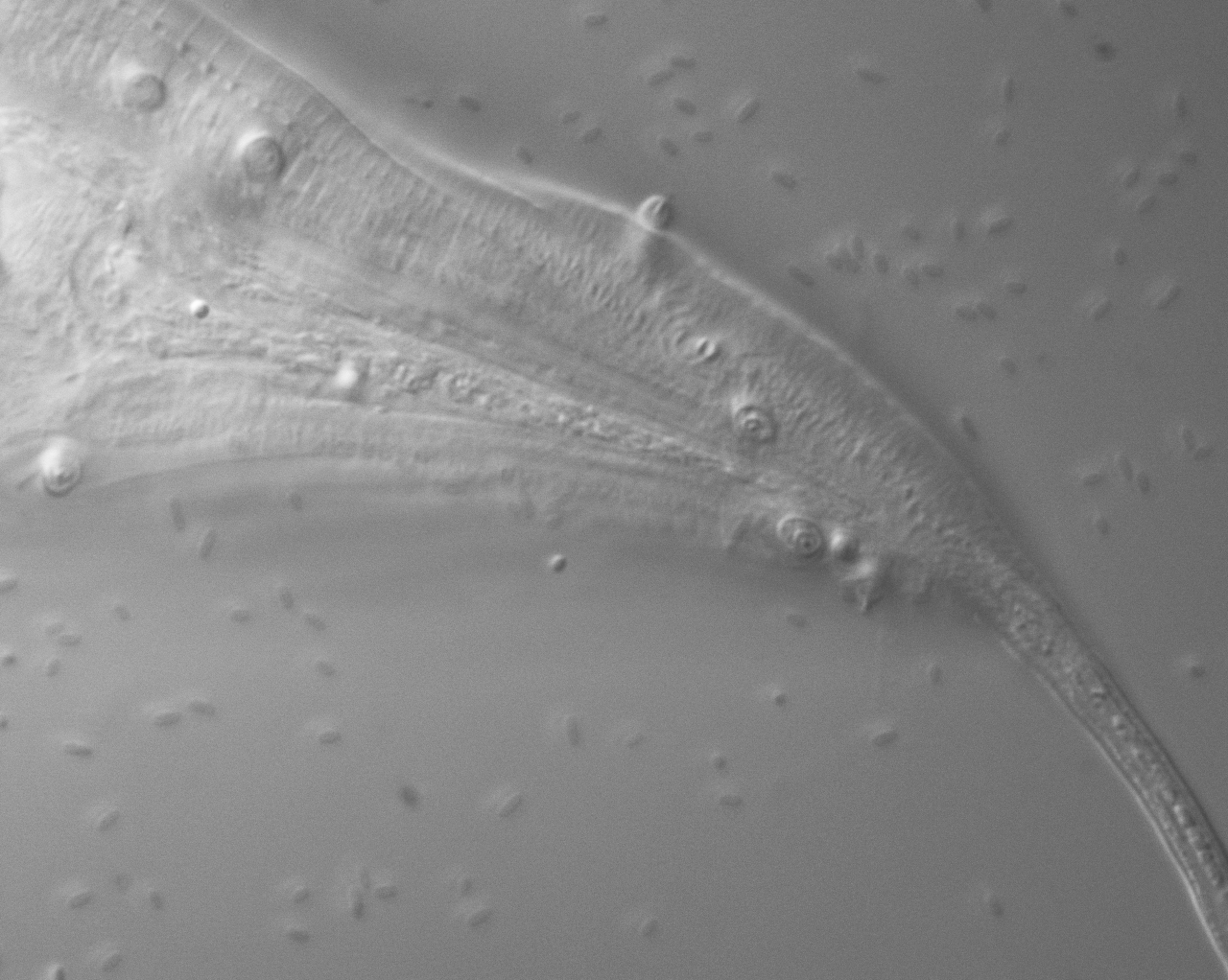

Supplement: S14 Fig — Diplogasteroides nasuensis strain SB335, adult male ablation result #18 (see Table 1). DIC micrograph. (TIF) [file pone.0348186.s014.tif]

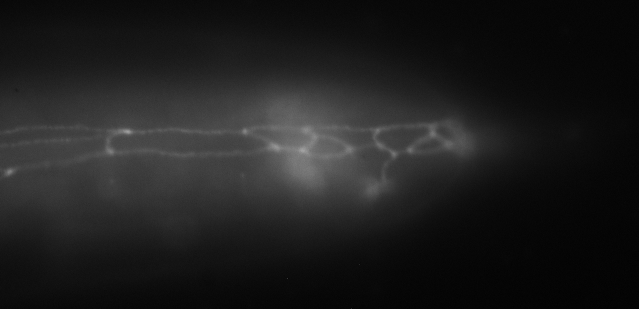

Supplement: S17 Fig — Poikilolaimus oxycercus strain EUK106 early L3 male, immunofluorescently stained with MH27 antibody. Epifluorescence micrographs as a z-stack, TIFF format. (TIF) [file pone.0348186.s017.tif]

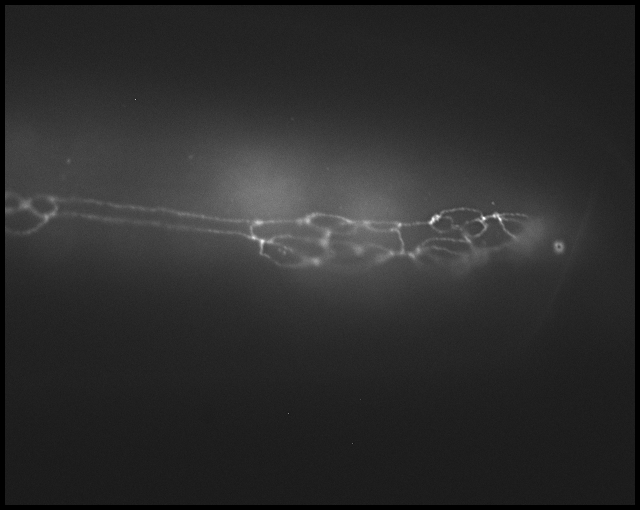

Supplement: S19 Fig — Poikilolaimus oxycercus strain EUK106 L4 (stage 1) male, immunofluorescently stained with MH27 antibody. Epifluorescence micrographs as a z-stack, TIFF format. (TIF) [file pone.0348186.s019.tif]

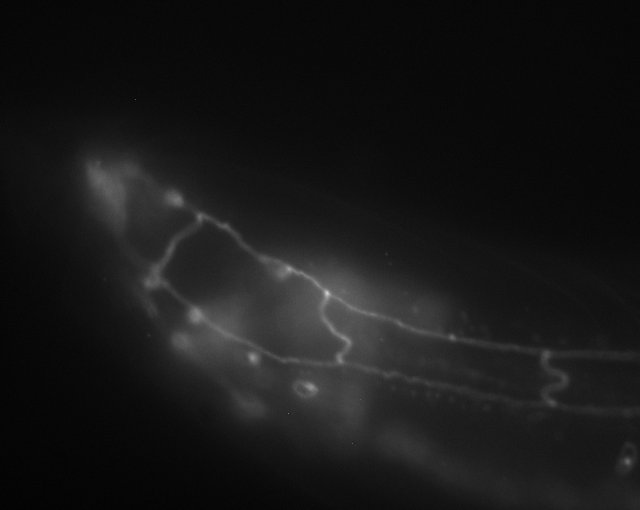

Supplement: S20 Fig — Poikilolaimus oxycercus strain EUK106 L4 (stage 3) male, immunofluorescently stained with MH27 antibody. Epifluorescence micrographs as a z-stack, TIFF format. (TIF) [file pone.0348186.s020.tif]

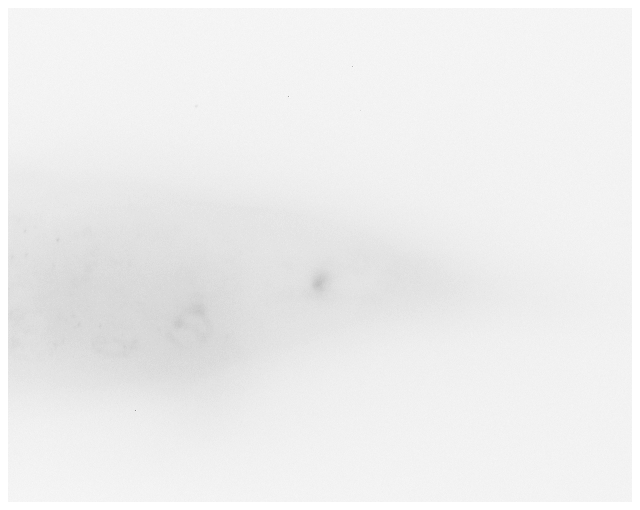

Supplement: S23 Fig — Haematozoon subulatum strain SB303 L4 (stage 1) male, immunofluorescently stained with MH27 antibody. Epifluorescence micrographs as a z-stack, TIFF format. (TIFF) [file pone.0348186.s023.tiff]

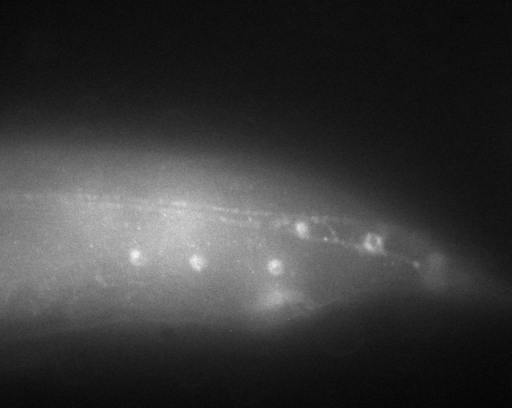

Supplement: S24 Fig — Haematozoon subulatum strain SB303 L4 (stage 3) male, immunofluorescently stained with MH27 antibody. Epifluorescence micrographs as a z-stack, TIFF format. (TIF) [file pone.0348186.s024.tif]

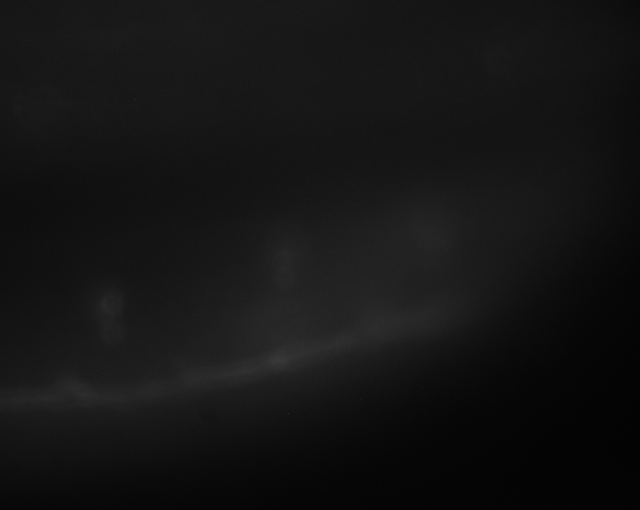

Supplement: S31 Fig — Brevibucca saprophaga strain SB261 L4 (stage 3) male, immunofluorescently stained with MH27 antibody. Epifluorescence micrographs as a z-stack, TIFF format. (TIF) [file pone.0348186.s031.tif]
