# Supplementary material for: Developmental constraints in the repeated evolution of male tail characters in rhabditid and diplogastrid nematodes
Source: PLoS One. 2026 Apr 28;21(4):e0348186. doi: 10.1371/journal.pone.0348186 (PMC13123947; doi:10.1371/journal.pone.0348186)
Supplement: Attachment — Submitted filename: ResponseToReviewers.docx [file pone.0348186.s034.docx]

**New York University**

*A private university in the public service*

David H. A. Fitch, Ph.D., Professor of Biology
Center for Developmental Genetics, Department of Biology
1009 Silver Center, 100 Washington Square East
New York, NY 10003
Tel.: (212) 998-8254
E-mail: david.fitch@nyu.edu

April 3, 2026

**RESPONSE TO EDITOR AND REVIEWER COMMENTS**

*Journal Requirements*

1. We believe the style and file naming system now follows the *PLoS ONE* guidelines.

2. Please add the following statement to our financial disclosure: "The funders had no role in study design, data collection and analysis, decision to publish, or preparation of the manuscript." Many thanks for changing the online submission form accordingly on our behalf, as you have offered.

3. We have removed the Acknowledgements section of the manuscript. Again, if this requires changing the online submission form on our behalf, we thank you for doing this as well. (We decided to remove the Acknowledgements altogether, because thanking the editor and reviewers is apparently also not allowed in this section, as is acknowledgement of funding sources.)

4. As requested both by the Editor and Reviewer #1, we have now included the raw data used for delineating the adherens junctions depicted in all figures. These files are in TIFF format, listed and described in a new Supplemental Information section, and uploaded as supplemental files S1_Fig.tif to S32_Fig.tif.

*Reviewer Comments to the Author*

Note: the line numbers referred to are those in the markup (Track Changes) copy of the manuscript.

Reviewer #1

1. We now clarify our use of the term "developmental constraint". Firstly, we delete all references to the term "canalization", the ontology of which is admittedly fraught in the literature. Canalization has been used to refer to evolutionary bias, but in developmental biology is more appropriately associated with the notion of restriction in cell fate potential trajectories. Because we view the terms "constraint" and "bias" as basically equivalent, we have also deleted most (but not all) uses of the term "bias".

Secondly, in the first paragraph to the Introduction, we try to clarify the difference in meanings of the terms "constraint" and "parallelism" and provide a clear operational definition that would allow us to recognize "degree of constraint" (e.g. "strong" vs. "weak" constraint). Specifically, parallelism is expected to be one result of constraint: i.e. because of constraints in the kinds of mechanisms accessible to evolution, we expect the same mechanisms to be used for producing similar characters in independent lineages (recurrent evolution). Thus, parallel evolution should be a good indicator for developmental constraint. Also, the greater the *degree* or frequency to which the same mechanism is used recurrently, the *stronger* must be the constraint.

Hopefully, this clarifies both the ontological and epistemological reasons for our approach and eliminates redundant terminology.

2. The reviewer suggested that "several key conclusions" are sensitive to differences that have been proposed to the tree topology, and that we should "explicitly discuss how these topology differences could affect the inferences of character evolution". Also, the reviewer suggested to better justify the Dollo criterion for resolving the tail tip evolution character changes.

We therefore revised several aspects to the MS:

First, we now point out that the phylogeny we are using has one ambiguous branching order near the base of the tree, but that nearly all other molecular studies and modern taxonomies resolve this ambiguity by keeping Bunonematidae within a monophyletic Rhabditina group, with Brevibuccidae as the sister taxon to Rhabditina (lines 266-273).

In lines 249-264, we now justify further our choice in using the more taxon-rich phylogenetic framework. Specifically, the sister taxon of Diplogastridae, *Rhabditoides*, is not included in the whole-genome phylogenetic studies and only in our study. *Rhabditoides* is quite robustly placed within rhabditids after the divergence of Pleiorhabditis. (Note that an additional taxon, *Haematozoon*, was also not included in the whole-genome studies and is important as a sister taxon to Pleiorhabditis, thus helping to place the diplogastrid lineage to diverge after Pleiorhabditis.) Besides the diplogastrid lineage, all other phylogenetic topologies are congruent between these studies (except that the other study also did not include a Brevibuccidae representative).

Even so, we tested the effect of the other phylogeny's placement of Diplogastridae on our inferences of character evolution. Contrary to the reviewer's suggestion, all our inferences but one are ***in***sensitive to different placements for diplogastrids. The *only* character evolution inference affected is that of whether the anterior phasmid position in Diplogastridae is interpreted as plesiomorphic vs. derived. We now make this caveat explicit in lines 317-338.

In lines 289-301, we now more explicitly justify our use of the Dollo criterion for resolving polarities for the tail tip evolution character changes. Specifically, gain of tail tip morphogenesis (TTM) is complex but loss of TTM is easy, as justified by several of our genetic, molecular and genomic studies in *C. elegans*.

We had already dealt with the two alternative interpretations for the two changes in GP number that occurred near the base of the phylogeny (lines 307-316), so we just rewrote this section to make it clearer. We also adjusted the relevant paragraph in the Conclusions section (lines 720-738).

3. The reviewer suggested that we be more explicit in stating the degree of uncertainty in the homology assignments of GPs for taxa in which they are inferred "indirectly" (we assume this means via our analysis of the AJ immunofluorescent staining patterns of where the GP cells originate relative to other cells in the lateral epidermis). We have indeed tried to be explicit in describing what is observed and how our inferences are derived from these observations; e.g. the inference that GP homolog v2 is the GP that is absent in *Brevibucca* and *Poikilolaimus*. For example, see lines 706-738.

4. The reviewer suggested we use more cautious phrasing with respect to conclusions from the laser ablation studies. We changed the phrase phasmid positions changes are "never" due to changes in lineage and "always" due to migration simply to "not" changes in lineage and "are" due to migration. In other cases, we note explicitly that some condition is present "in all species tested" (e.g. lines 810-812, 839).

The reviewer also noted that in some taxa, the "number of informative ablations are small". All of the ablations listed in Table 1 are informative. Perhaps the reviewer is referring to the "nothing" entries for the females, meaning that "no phasmid or male structure was affected"? In other words these "nothing" results are indeed informative because the show that a cell ablation did not delete a structure, so that cell was not a precursor of such structures/organs. Also, we argue that, because all our ablations yielded consistent results, and because rhabditids have almost entirely invariant cell lineages, even a small number of successful ablations will be informative. Certainly, the number of our ablation experiments (which are technically quite challenging) are on par with or exceed the famous ablation studies in rhabditids performed by other labs. In addition, we have now added "raw image data" documenting how we identified cells for ablations and the results of ablations on adult organs (S1-S16 Figs).

*Minor Comments on Clarity and structure*

We tried to clean up the language (and typos) to make it as readable as possible—see the markup version of the manuscript. However, we do think it is important to preserve the detailed descriptions of our results. We hope that the Discussion section helps to put all these results into a meaningful context in a way that is digestible.

*Figures 7-10*

The reviewer suggested that we add "simplified schematics" to reduce the complexity of the adherens junction staining results. We argue that we already do this in Fig. 1, which shows the "archetype" (actually a simplified schematic), the fundamental structure of which is completely conserved among all these species. To make the correspondence between this archetype and the AJ pattern reconstructions for each species explicit, we used consistent color coding of the different parts of this archetype. We now also provide the raw data underpinning these reconstructions as supplemental files (S17-S31 Figs). Thus, we believe we have made our best "good faith" effort to present our data in as rigorous but understandable a fashion as possible.

Reviewer #2

1. There is no molecular analysis performed in this study, so we do not include such a subsection in the methodology section. As mentioned in the text, the molecular phylogeny used was already published (Kiontke et al., 2007) with taxa renamed according to Sudhaus (2011 and following).

2. The number of organisms investigated by laser ablation are noted in Table 1. The number of animals investigated for AJ reconstructions is not given, because there were many observations and literally hundreds of worms were stained. Because the reconstructions are very tedious and time-consuming, we only picked the samples shown in Figs 7-11 as representative of the many observations. For completeness, we now additionally provide the raw data underpinning these reconstructions (i.e., the fluorescent microscopy image *z*-stacks).

3. As per the PLoS ONE instructions to authors, a Conclusion section is optional. We did not see the need to add a Conclusion section, since the final two paragraphs of the Discussion section already offer the paper's concluding remarks (explicitly), and we reasoned that such an addition would be unnecessarily redundant.

4. The reviewer asked what the "reason" was for choosing this topic of research. We believe the first paragraphs of the Introduction serve to clarify this. We additionally cite a representative sampling of references for context should any reader wish to delve further into these longstanding and important questions of developmental constraint in evolution.

5. The reviewer asks us to "elaborate our hypothesis". We believe we have already provided our hypotheses. For example, one of them is explicitly depicted in Fig. 1 (actually alternative hypotheses, their predictions, and the results of the tests of these hypotheses are explicitly considered in the paper).

6. We have tried to provide complete explanations of all abbreviations used throughout the manuscript. Note that the abbreviations for the GP homologies designated in the figures are all explained in the text, depicted in Fig. 1, and used consistently throughout the manuscript. We therefore thought it would be unnecessarily redundant to repeat what these abbreviations mean in the legends to every figure.

7. The legend for Fig 6B already noted that the asterisk (*) shows the missing feature resulting from the laser ablation (similar to how an asterisk is used in the other laser ablation figures, e.g. Fig 4).

8. The reviewer asks about the "molecular markers or characters that have been used to deduce the phylogenetic tree". Although we do state that this is a molecular phylogeny, we also note that it was taken from previous studies, which are cited here. Instead of reiterating those analyses in this paper, we feel that interested readers can refer directly to the original papers if they need more information about the characters and analyses used to infer the phylogeny (esp., Kiontke et al., 2007). Here, we utilize these already-determined phylogenies to explore character evolution, but we do *not* deduce anew the phylogeny in this paper.
